# Effect of N-Acetylcysteine on initial Carious Enamel Lesions in primary teeth: an In-vitro study

**DOI:** 10.1186/s12903-023-03224-3

**Published:** 2023-07-25

**Authors:** Shaimaa M. Mahfouz Omer, Randa H. El-Sherbiny, Shaimaa S. EL-Desouky

**Affiliations:** 1 Pediatric Dentistry, Preventive Dentistry, and Dental Public Health Department, Faculty of Dentistry, Suez Canal University, Ismailia, Egypt; 2 Oral Pathology Department, Faculty of Dentistry, Suez University, Suez, Egypt; 3 Pediatric Dentistry, Oral Health and Preventive Dentistry Department, Faculty of Dentistry, Tanta University, Tanta, Egypt

**Keywords:** N-Acetylcysteine, Carious Enamel Lesions, PH cycling, Primary teeth

## Abstract

**Background:**

Dental caries initiates with non-cavitated enamel lesions as the first stage. The cariogenic potential of N-Acetylcysteine (NAC) may be due to its usage frequency and form. This study aimed to evaluate the impact of exposure time of NAC on initial enamel caries-like lesions in primary teeth by assessing the morphological alteration using a scanning electron microscope (SEM) and mineral content using energy dispersive x-ray spectroscopy (EDX).

**Methods:**

Forty primary incisor teeth were randomly divided into 4 groups S, S1, S2, and S3 (10 specimens/group). Teeth crowns were cut from their roots and inserted into an acrylic mold with its buccal surface directed upward. Centrally isolated enamel window (2 × 2 mm) on the tooth was done. Ten specimens were selected to evaluate normal enamel while the remaining thirty specimens were immersed in demineralizing solution for 96 h to produce enamel caries-like lesions. PH cycling was performed by immersing each tooth sample in 20 mL of demineralizing solution for 3 h then, preserved for the remaining day hours in 10 ml of artificial saliva interspersed with treatments applications with 10 ml NAC for 10 min twice a day for one- or three-months different treatment modalities. Thermocycling was done for all specimens then they were subjected to SEM and EDX analysis. ANOVA and Bonferroni post hoc tests were utilized in data analysis.

**Results:**

In teeth treated by NAC for 3 months (group-S3), SEM images showed severe loss of enamel architecture with large NAC deposits detected. A meaningful difference was observed among different groups concerning calcium, phosphorus, fluoride, ca/P ratio, carbon, nitrogen, and oxygen contents (P < 0.05).

**Conclusion:**

NAC had a detrimental impact on enamel caries-like lesions in human primary teeth.

## Introduction

N-acetylcysteine (NAC), a precursor of the antioxidant glutathione (GSH), is among the most promising naturally- derived antioxidant compounds with an outstanding safety profile. It is extracted specifically from the Allium plant family whose thiol group effectively hunts down reactive oxygen species (ROS) and promotes the production of GSH [1]. NAC is a mucolytic drug that has been used for a long time to decrease the mucous viscosity in pulmonary compromised patients, involving children with cystic fibrosis through inhalation, oral, and intravenous routes [2]. Additionally, it is greatly useful in inhibiting severe liver injury after paracetamol overdose if given within 8–10 h as it averts GSH depletion, reduces hepatocyte injury, and reverses the oxidation and arylation of critical hepatic proteins and enzymes [3]. It has also been documented that NAC inhibits osteoclast activity, which further prevents bone resorption [4]. Furthermore, NAC as an antioxidant agent has been used as an adjuvant and/or preventive treatment against SARS-CoV-2 [5].

Dental caries is one of the most prevalent illnesses among children, affecting over 530 million children globally which is a cyclical process that alternates between periods of demineralization and remineralization. When demineralization prevails over remineralization, carious lesions occur which affect the tooth minerals promoting rapid dentin breakdown [6]. Dental caries increased during the COVID-19 pandemic among children due to their altered lifestyles to be more cariogenic with increased sugar consumption [7] also, postponed dental care, and the consumption of COVID-19 medications which causes xerostomia [8, 9].

There is limited research on the relationship between NAC and dental caries. Limited studies have shown that NAC may have some benefits for oral health, such as reducing plaque formation by inhibition of multi-species biofilm formation on hydroxyapatite [2]. The potential anti-caries benefit of NAC may be directly related to reducing the biofilm coverage which reduces the degree of acid generation and the amount of time that the surface is exposed to a lower pH [2]. However, more research is needed to confirm this benefit and to determine the long-term effects of NAC on teeth’s enamel.

Primary and permanent enamel are morphologically and histologically distinct, implying that primary teeth are more vulnerable to demineralization. primary teeth’ prisms are smaller and more curved also, their hydroxyapatite crystals differ significantly. Enamel crystals in both primary and permanent teeth are imperfect kinds of hydroxyapatite, which is primarily composed of calcium (Ca^2+^), phosphate (PO_4_^3−^), and hydroxyl (OH^−^) ions with some ‘impurity’ ions, such as fluoride (F−), carbonate (CO_3_^2−^) and sodium (Na^+^), in a crystalline structure with simplified chemical formula: Ca_10_–Na_x_(PO_4_)_6–y_(CO_3_)_z_(OH)_2_^–^_u_F_u_. Carbonate (CO_3_^2−^) is an important impurity ion in the distinction between primary and permanent enamel. When CO_3_^2−^ exists in the apatite crystal, it generates a deformed lattice structure and makes the crystals more soluble than the balanced hydroxyapatite. As the primary enamel has a higher amount of CO_3_^2−^, it is more prone to breakdown. In addition, it is less mineralized and more porous with higher organic content and, consequently, lower elasticity and lower surface microhardness than permanent enamel [10].

The cariogenic ability of any medication accounts for its sugar content as well as its dosage, frequency, and usage form. Pharmaceutical companies use some types of sugar in the compositions of numerous pediatric medications to disguise the flavor of their active constituents [11]. The purpose of this study was to assess the impact of exposure time of NAC on artificially produced early enamel caries-like lesions in primary teeth by assessing the morphological alteration utilizing scanning electron microscope (SEM) and mineral content using energy dispersive x-ray spectroscopy (EDX).

## Materials & methods

### Study setting and ethical consideration

A controlled in-vitro study had been carried out at the Pediatric Dentistry Department, Faculty of Dentistry, Suez Canal University after obtaining the approval of the Research Ethics Committee (REC), Faculty of Dentistry, Suez Canal University (Code 587/2022) in accordance with the ethical guidelines outlined in the 1964 Helsinki Declaration and its subsequent revisions. Informed written consent from parents was attained to use their children’s extracted teeth in the research.

### Eligibility criteria

**Fig. 1:**
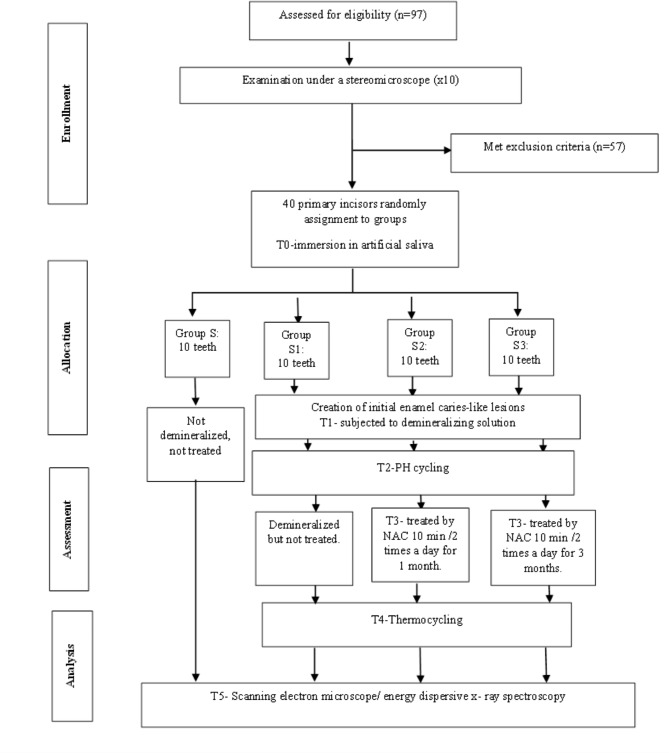
Schematic representation of experimental study design

Ninety-seven extracted primary incisors due to shedding were gathered from the Pediatric Dentistry Department’s outpatient clinic at the Faculty of Dentistry, Suez Canal University. The inclusion criteria of selected teeth were sound, free of white spots or cracks, and not previously restored. However, teeth with any cracks, stains, or other flaws were excluded after inspection underneath a stereomicroscope (x10). Figure (1) denotes a flow chart that includes enrollment, allocation, assessment, and sample size analysis.

### Sample size calculation and randomization

G*Power (version 3.1.9.2, University Kiel, Germany. Copyright (c) 1992–2014) [12] was utilized to calculate the sample size. The estimated sample size (n) for this study should be 40 samples (teeth) based on the effect size d of 0.73, alpha (α) level of 0.05, and Beta (β) level of 0.05, i.e., power = 95%.

Simple randomization was computer generated by the Research Randomizer software program (https://www.randomizer.org/) [13]. An independent person put randomization codes in sequentially numbered, secured, opaque wrappers to ensure covert distribution into the four groups.

### N-Acetylcysteine

The concentration of 200 mg in the form of effervescent sachets N-Acetylcysteine (Acetylcystein eff. Instant sachets®, Sedico Pharmaceutical Co., 6 October city, Cairo, Egypt) was used in this study. NAC solution was daily freshly prepared for each sample by dissolving one sachet completely in 120 ml of water. The pH value of the prepared NAC solution was calculated with a digital pH meter (Qingdao Tlead International Co., Ltd. Shandong, China); it was measured three times in the Chemistry Department, Science faculty, Suez University then, the mean value was calculated. The mean pH value was 4.79 which was checked continuously at each time of specimen immersion.

### Specimens’ grouping

All teeth specimens were randomly divided (10 specimens/group) according to different treatment modalities:

- **Group-S: (negative control)** (*n* = 10, normal enamel specimens).
- **Group-S1: (positive control)** (*n* = 10, demineralized enamel specimen which had initial enamel caries-like lesions).
- **Group-S2: (experimental)** (*n* = 10, demineralized enamel specimens) which were treated by NAC 10 min /2 times a day for 1 month.
- **Group-S3: (experimental)** (*n* = 10, demineralized enamel specimens) were treated by NAC 10 min /2 times a day for 3 months.

### Tooth specimen preparation

Forty eligible teeth were cleaned of debris, blood, and soft tissue and then disinfected with 0.1% thymol solution [14]. The teeth samples were kept at 37 °C in weekly-changed deionized water (SIGALD, Sigma-Aldrich Chemie GmbH) for a maximum of one month until use [15]. Teeth crowns were cut from their roots at the cementoenamel junction using a micro saw with 0.6 mm disc thickness (ISO MET 4000, Buehler, USA) at a speed of 2500 rpm and feeding rate of 10 mm /min using copious water coolant. Each tooth specimen was fixed in an acrylic mold with its buccal surface directed upward. About 150 μm enamel layer was eliminated using sequential polishing with 600 and 800-grit silicon carbide polishing stripes [16]. Pieces of 2 × 2 mm modeling wax sheet (Perfect Wax Base Plate, Turkey) were positioned in the buccal surfaces’ middle third, followed by two coats of transparent acid-resistant nail polish implemented all around the crown surface and allowed to dry. Then, the wax piece was removed, revealing a clean, centrally isolated enamel window (2 × 2 mm) on the tooth surface [16]. All specimens were incubated at 37 ^o^C in artificial saliva ((KCl 0.12%, NaCl 0.08%, MgCl_2_ 0.01%, K_2_Hpo_4_ 0.03%, CMC-Na 0.1% (sodium carboxy Methyl cellulose), CaCl_2_ 0.01%, Sterile deionized water 99.6%, (pH = 7)) throughout the steps the study [17].

### Creation of initial enamel caries-like lesions

Ten specimens were randomly selected to evaluate normal enamel surfaces while the remaining thirty specimens were dipped in a bath containing demineralizing solution to initiate enamel caries-like lesions. Each specimen had been exposed to 20 ml of demineralizing solution (2.2 mMol CaCl_2_, 2.2 mMol Na_3_Hpo_4_, 0.05 mMol acetic acid, 1 mMol KOH (pH 4.2)) [18, 19] in a separate capped-test tube for 96 h, in an incubator at 37 °C [20]. The demineralizing solution was formulated in the Chemistry Department, Faculty of Science, Suez University; its pH was measured continuously by a digital pH meter & adjusted to 4.2 using 1 mMol potassium hydroxide [16]. The solution was freshly prepared and changed daily to avoid supersaturation.

### PH cycling

Each tooth specimen was soaked individually for 3 hours in 20 mL of the demineralizing solution and then, preserved in 10 ml of artificial saliva for the remaining day hours interspersed with treatment applications (18, 21, 22). Regarding the experimental groups, distilled water was used to wash each tooth specimen for 10 seconds and then, treated separately with 10 ml NAC for 10 min (23) two times per day (pre-and post- the demineralizing period) for one or three months according to each group (18, 24).

### Aging of the specimens

All specimens were exposed to a thermocycling aging procedure for 1000 cycles with a dwell period of 25 sec, a transport time between cycles of 10 sec, and at a temperature range of 5–55°C (15) at the Fixed Prosthodontics Lab, Faculty of Dentistry, Suez University, to appropriately replicate the oral cavity environment.

All Samples were removed from artificial saliva to dry before analysis for 2 hours then, samples were dried with ethanol solution and sprayed with gold in a vacuum evaporator. The morphology of the specimen surface was explored with a scanning electron microscope (Quanta FEG − 250, National Research Center, Dokki, Egypt) in high-vacuum mode at 20 kV with 2000X, 6000X magnification. Then, all specimens were exposed to energy dispersive x-ray spectroscopy (Quanta FEG-250 model AMETEX, National Research Center, Dokki, Egypt) to quantity the values of calcium, phosphorous, fluoride, carbon, nitrogen, and oxygen contents (weight %) also, calcium-phosphorous ratio (weight%) was calculated. Both SEM imagers and the examiner (second author) were blinded to different treatment modalities.

### Statistical analysis

Data were gathered, tabulated, and statistically analyzed using SPSS software for Windows, version 26.0 (Statistical Package for Social Science, Armonk, NY: IBM Corp). The normality test (Shapiro-Wilk) was used at a significant level of alpha = 0.05 and it was not significant for all variables; this means that the data was normally distributed. Mean and standard deviation (SD) were used to calculate descriptive statistics. Comparison between groups was done using one-way ANOVAs while pairwise comparisons were made using Bonferroni post hoc tests. The level of significance was set as p-value ≤ 0.05).

## Results

### Scanning electron microscope (SEM)

**Fig. 2:**
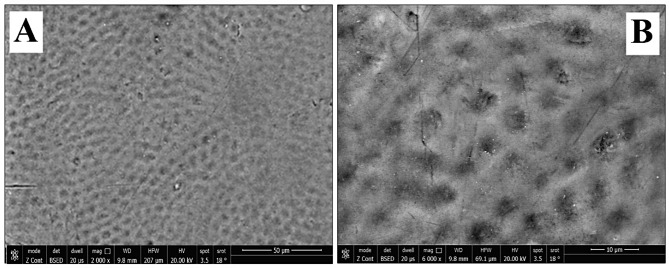
**A**&**B** SEM photographs showed sound enamel with a smooth surface and normally arranged enamel rods with inter-rod material producing a keyhole appearance. Small pits and cracks are found. (Magnification 2000X, 6000X respectively)

**Fig. 3:**
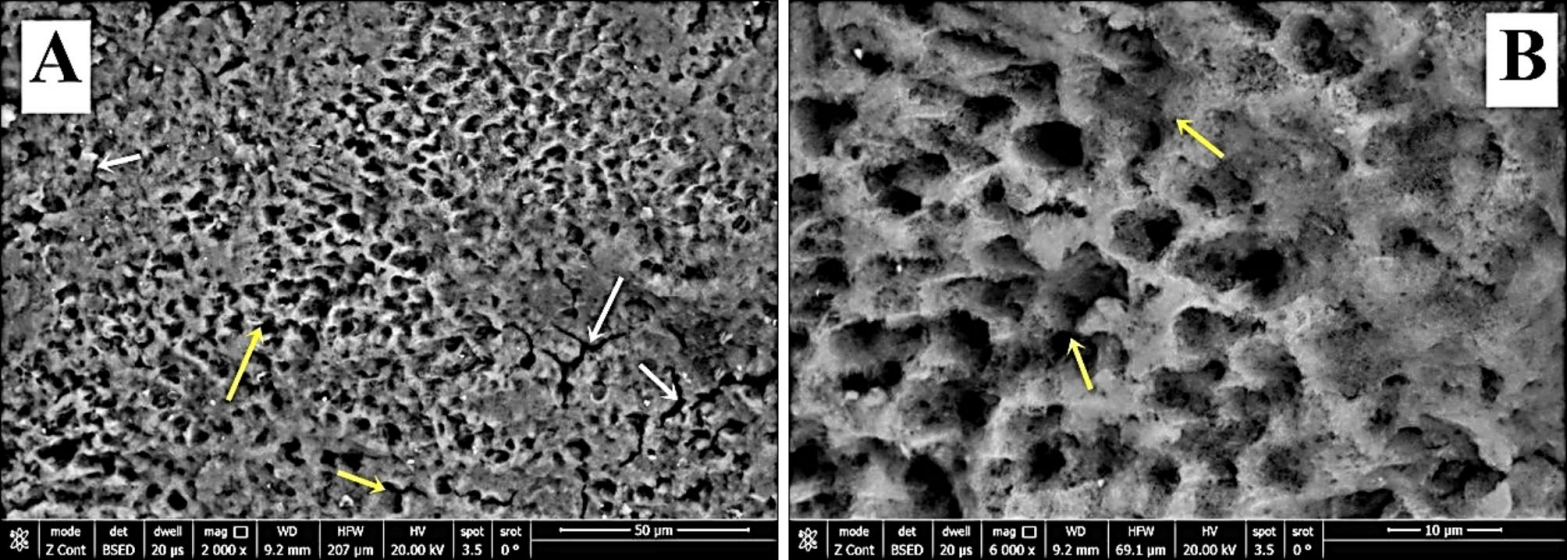
**A**&**B** SEM photographs of group-S1 showed an irregular enamel surface, numerous porosities with the destruction of the interprismatic substance (yellow arrows), and the presence of multiple cracks (white arrows). (Magnification 2000X, 6000X respectively)

**Fig. 4:**
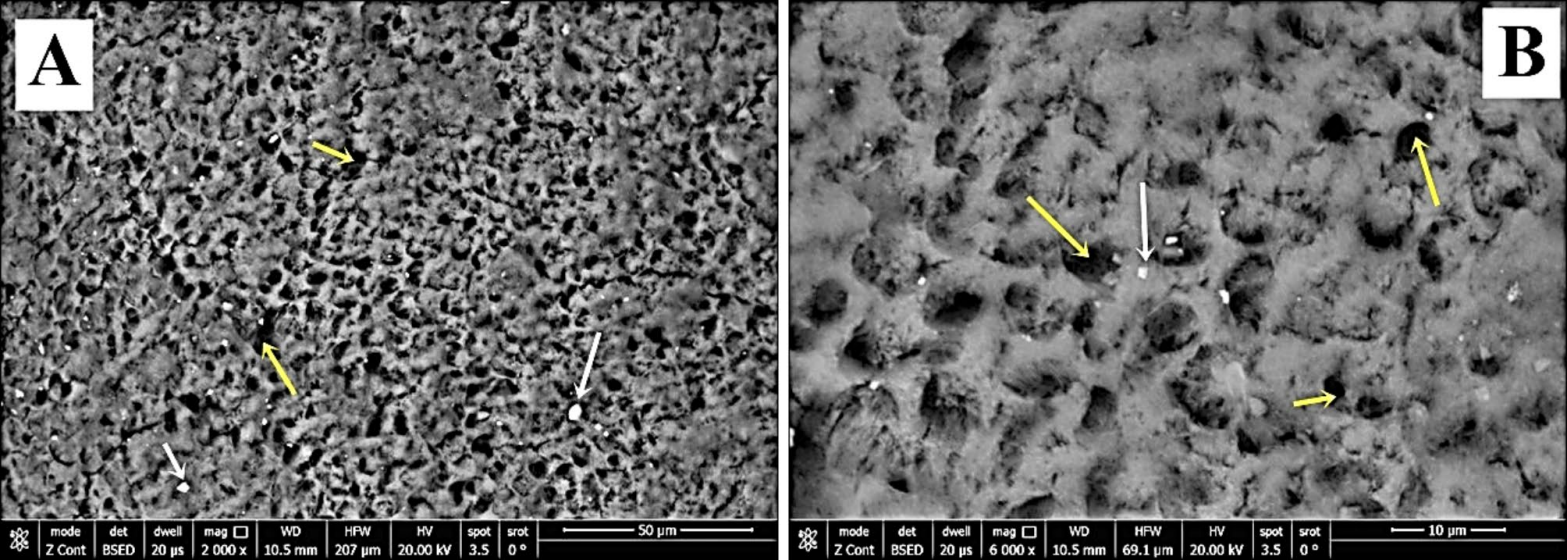
**A**&**B** SEM of group-S2 showed irregular surface with interprismatic dissolution (yellow arrows) and NAC deposits on the surface (white arrows) (Magnification 2000X, 6000X respectively)

**Fig. 5:**
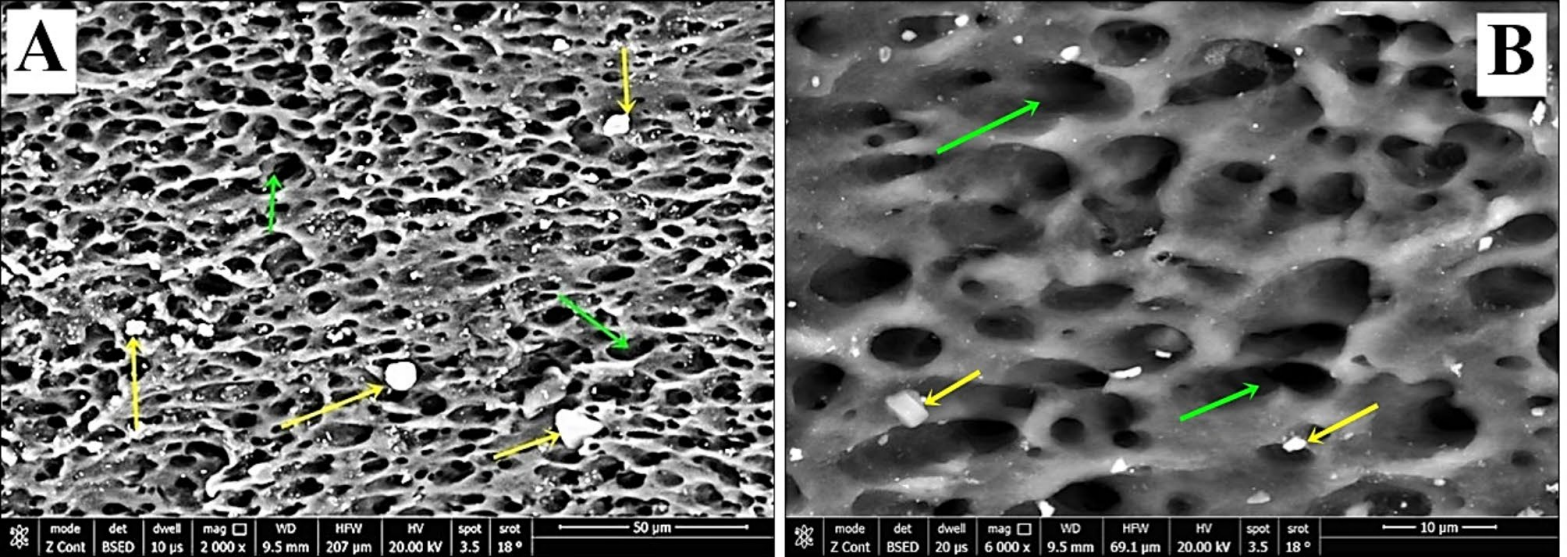
**A**&**B** SEM photographs of group-S3 showed an irregular surface of enamel with the presence of large pores and severe dissolution of the interprismatic substance (green arrows). Large NAC deposits were incorporated into the demineralized surface (yellow arrows). (Magnification 2000X, 6000X respectively)

Regarding group-S (negative control), SEM images demonstrated a smooth intact enamel crystalline structure with a normal keyhole appearance and few minute depressions (Fig. 2a&b). While, in group-S1(positive control), SEM images showed that the enamel surface was porous and the enamel prisms’ structural integrity was compromised, with the destruction of interprismatic material and partial loss of normal keyhole appearance with the evidence of a collapse in enamel rods (Fig. 3a&b). SEM images revealed irregular enamel surface with loss of keyhole appearance in teeth specimens treated by NAC for one month (group-S2). Moreover, in the same group, some areas showed dissolution of interprismatic substance with few deposited NAC particles on the boundary of the prism sheath and the interprismatic substance (Fig. 4a&b). In teeth treated by NAC for 3 months (group-S3), SEM images disclosed severe loss of enamel architecture with severe porosities and dissolution of the apatite crystals inside the prisms as well as the destruction of interprismatic structure that all characterize severe enamel demineralization. Large NAC deposits were detected (Fig. 5a&b).

### Energy dispersive X-ray spectroscopy (EDX)

**Fig. 6:**
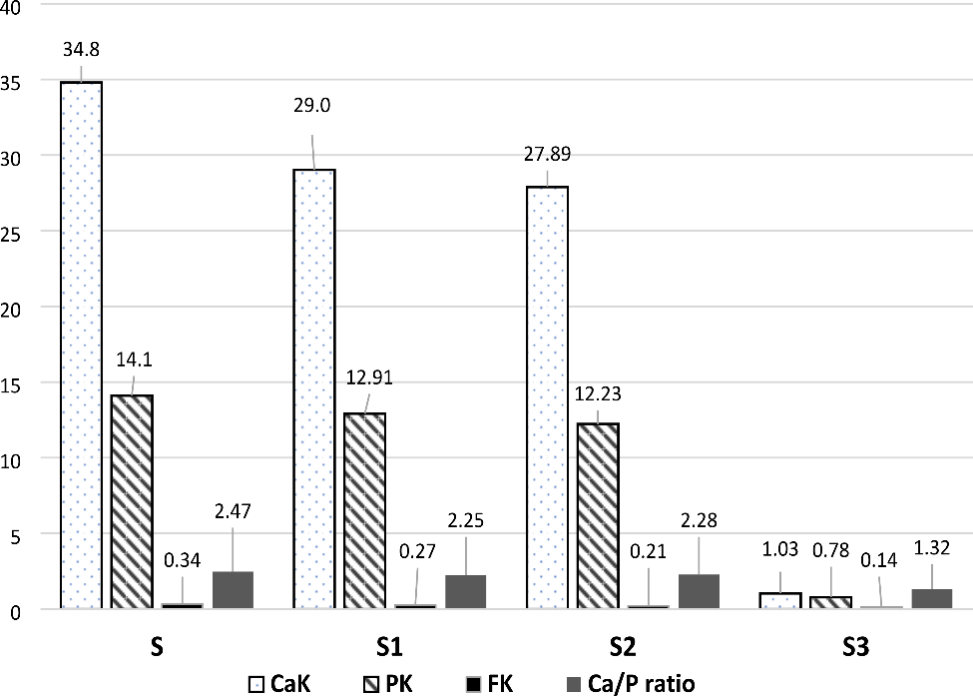
Bar graph presenting the average Ca K, PK, FK, and Ca/P ratio values (wt.%) through the study steps

In relation to fluoride content, the pairwise comparison displayed a meaningful difference between all groups. Group-S had the highest mean value (0.34 ± 0.04) followed by group-S1 (0.27 ± 0.03) however group-S3 had the lowest content (0.14 ± 0.01) (Table-1, figure-6). The pairwise comparison showed a significant decrease in phosphorus content among different groups except between group-S1& -S2 (P = 0.44). Group-S3 had the greatest phosphorus decrease with an average value of 0.78 ± 0.04. Regarding calcium content, group-S1 showed a significant decrease after demineralization than group-S (normal enamel) (29.03 ± 1.35, p < 0.001). Also, there was a significant statistical reduction in calcium content in group-S2 & -S3 in comparison with group-S after one month & three months of treatment with NAC respectively. While a non-significant decrease was found between group-S1 & -S2 (p = 0.974). Concerning the calcium-phosphorus ratio, there was a significant decrease between group-S3 and other different groups (p < 0.001). While a non-significant difference was observed between group-S, -S1, and -S2. The highest mean value of calcium-phosphorus ratio was presented in group-S (2.47 ± 0.12) while group-S3 was the lowest one (1.32 ± 0.17).

**Table 1:** Weight percentages of calcium, phosphorus, fluoride, and Ca/P ratio in different groups

Groups	CaK Mean ± SD	PK Mean ± SD	FK Mean ± SD	Ca/P ratio Mean ± SD
Group-S	34.80 ^a^ ± 1.79	14.10 ^a^ ± 0.15	0.34 ^a^± 0.04	2.47^a^ ± 0.12
Group-S1	29.03 ^b^ ± 1.35	12.91 ^b^ ± 0.62	0.27 ^b^±0.03	2.25^a^ ± 0.13
Group-S2	27.89 ^b^ ± 1.99	12.23 ^b^ ± 0.73	0.21 ^c^± 0.01	2.28 ^a^ ±0.12
Group-S3	1.03 ^c^ ± 0.12	0.78 ^c^ ± 0.04	0.14 ^d^ ±0.01	1.32^b^ ± 0.17
P-value	0.001**	0.001**	< 0.001**	0.001**
Multiple Comparisons using Bonferroni post-hoc				
S vs. S1	< 0.001**	0.03*	< 0.001**	0.46
S vs. S2	< 0.001**	< 0.001**	< 0.001**	0.48
S vs. S3	< 0.001**	< 0.001**	< 0.001**	< 0.001**
S1 vs. S2	< 0.001**	0.44	0.974	0.829
S1 vs. S3	< 0.001**	< 0.001**	< 0.001**	< 0.001**

**, and different superscript letters indicates significance at P < 0.05

**Fig. 7:**
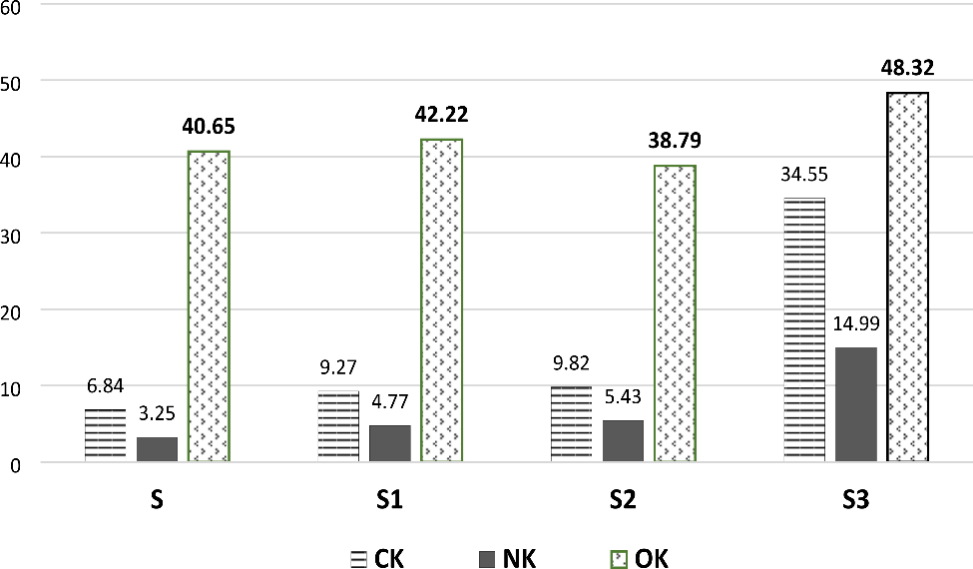
Bar graph explaining the average CK, NK, and ok values (wt.%) through the study steps

Table-2 showed clearly significant differences between different groups concerning carbon, nitrogen, and oxygen contents (P < 0.05). Regarding carbon content, group-S3 showed a significant increase after three months of treatment with NAC to other groups (0.34.55 ± 0.77, p < 0.001). Also, there was a statistically substantial increase in carbon content in group-S1& -S2 compared to group-S. While a non-significant increase was found between group-S1 & -S2 (p = 1.00) (figure-7). There was a significant statistical increase in nitrogen content between different groups (P < 0.05) except between group-S & -S1(P = 0.51). The highest mean nitrogen value was found in group-S3 (14.99 ± 1.48) followed by group-S2 (5.43 ± 0.69) while group-S revealed the lowest one (3.25 ± 0.41). In relation to oxygen content, group-S3 had a statistically significant increase compared to other groups with a mean value of 48.32 ± 2.29. Also, the pairwise comparison showed a non-significant difference between group-S, S1, and S2.

**Table 2:** Weight percentages of carbon, nitrogen, and oxygen in different groups

Groups	CK Mean ± SD	NK Mean ± SD	OK Mean ± SD
Group-S	6.84 c ± 0.65	3.25 ^c^±0.41	40.65 ^b^ ±2.52
Group-S1	9.27 ^b^ ± 0.57	4.77 ^c^± 0.51	42.22 ^b^±3.12
Group-S2	9.82 ^b^ ± 0.57	5.43^b c^± 0.69	38.79 ^b^±5.02
Group-S3	34.55 ^a^± 0.77	14.99 ^a^ ± 1.48	48.32 ^a^±2.29
P-value	< 0.001**	< 0.001**	< 0.001**
Multiple Comparisons using Bonferroni post-hoc			
S vs. S1	< 0.0001**	0.51	0.987
S vs. S2	< 0.0001**	0.04*	0.942
S vs. S3	< 0.0001**	< 0.0001**	0.04*
S1 vs. S2	1.00	< 0.0001**	0.927
S1 vs. S3	< 0.0001**	< 0.0001**	0.003**
S2 vs. S3	< 0.0001**	< 0.0001**	< 0.0001**
**, and different superscript letters means significance at P < 0.05			

## Discussion

N-acetylcysteine in the effervescent form is acidified with citric acid and sweetened with lactose so, its regular use raises the possibility of enamel dissolution and developing caries because of erosion (25). The NAC pH value was acidic after its measurement with a pH meter; this coincided with Neves et al., (26) and Pushpanjali et al., (27) who deduced that many pediatric medications showed an acidic pH. So, the present study aimed to assess the impact of exposure time of NAC on artificially produced initial enamel carious lesions by assessing the morphological alteration with scanning electron microscope (SEM) and mineral content using energy dispersive x-ray spectroscopy (EDX). These high-energetic devices detect micromorphological changes by magnifying & scanning the target region without causing any damage as they need little or no specimen preparation (14).

This in-vitro study was conducted because of its low cost and to minimize the effect of mystifying variables. Extracted primary teeth of the same tooth type were selected to standardize enamel thickness and tooth size, while the middle third of the buccal surface was chosen to lessen the surface enamel variation that may react differently to acid [28]. In addition, teeth were stored in Weekly-renewed deionized water for a maximum of one month at room temperature to avoid dehydration and alteration of minerals content, moreover, weekly deionized water change was made to avoid the growth of the bacteria.; this agreed with Amin et al., [23] and Tulumbaci & Gungormus, [29]. All teeth specimens were disinfected by thymol solution as the storage solution affected how the enamel components reacted to the used pH cycling model; this agreed with Soares et al.,[30] and Amaechi et al.,[31]. Moreover, 2% formaldehyde wasn’t selected for teeth disinfection in this study; this coincided with Moura. et al., [32] who confirmed that samples preserved in formaldehyde showed a high resistance to demineralization as a result of formaldehyde’s ability to fix proteins to the teeth surface. Teeth specimens were kept at 37 °C in the artificial saliva in an incubator throughout the steps of the study to resemble the oral circumstances [33].

Teeth specimens were polished for approximately 150 μm to remove the aprismatic surface enamel layer with markedly high mineral content which could interfere with the artificial demineralization process; this agreed with Salma et al., [16]. Initial enamel caries-like lesion was selected to be examined because, under clinical conditions, it would not likely be discovered or considered disputed. It was created by keeping the specimens for 96 h at 37 °C in the demineralization solution to produce a subsurface demineralization with an undamaged surface mimicking an initial enamel lesion.; this agreed with El-Gar et al., [20].

The PH cycle was selected to imitate the normal dynamic changes that occur inside the mouth involving the demineralization and remineralization processes; the pH cycling model utilized in the current study was constructed by Featherstone et al.,[22] and adjusted by Argenta et al., [18], also, the composition of the demineralizing solution and artificial saliva was like that utilized by Buzalaf et al.,[19]. PH of the used solutions was measured daily also; Fresh solutions were used to prevent the risk of reaching the saturation limit.

SEM pictures of group-S showed a smooth aprismatic layer which was affirmed by the EDAX testing that revealed high Ca, P, F, and Ca/P ratio than other groups with a statistically significant difference; this coincided with Jälevik et al., [34] and Akasapu et al., [35]. This can be clarified by the distinct physicochemical characteristics of sound enamel because of the greater hydroxyapatite content, the aligned configuration of extended apatite crystals into enamel prisms, and the intertwined orientation of vertical prisms in a three-dimensional picket-paling-like arrangement. All previous properties yield a biological material with exceptional physical resilience and hardness [36].

While SEM images in group-S1 revealed a rough, porous enamel surface that can be justified by the disposal of inorganic substances from the enamel and the superficial dissolution of the surface enamel. This was affirmed by the EDX assessment which recorded a substantial reduction in Ca, P, F, and Ca/P ratio after demineralization. This agreed with Kamath et al., [37] who stated that the demineralization of enamel causes hydroxyapatite dissolution and Ca/P ions diffusion towards the enamel surface, resulting in nearly150 µ width subsurface decalcification with an undamaged surface, mimicking an initial enamel caries. Also, this was in line with Tsai et al., [38] who used the high-resolution optical coherence tomography system for illustrating the acidic environment effect on the enamel topology and noticed an increase in the dispersive coefficient due to hydroxyapatite dissolution which resulted in surface irregularities.

The current EDX analysis demonstrated a substantial decrease in Ca, P, F, and Ca/P ratio in group-S2 which was presented as irregular surfaces with interprismatic dissolution in SEM images. This could be explained by NAC’s low pH (pH = 4.79). The threshold of supersaturation of calcium and phosphorus decreases as the pH decreases, and accordingly, the demineralization risk rises. Although there isn’t a precise pH level upon which demineralization commences, it is assumed that a general pH range of 5.5–5.0 is requisite for tooth minerals to dissolve [39]. This agreed with Jeong et al., [40] who reported that medication with a low pH as well as high citric acid levels has an affinity for the tooth enamel, causing enamel erosion. Moreover, Rytömaa et al. [41] specified that tooth enamel dissolves when a pH value equals 5.5, and acidic food with pH < 4 is most probably causing erosion. Furthermore, Lussi & Schaffner [42] disclosed that dental erosion is triggered by consuming acidic foods and O’Sullivan & Curzon [43] asserted that the risk of dental erosion is rising in many children who drink acidic beverages.

By treating the initial carious enamel surfaces in group-S3 with NAC for three months, SEM demonstrated additional enamel prism loss, resulting in a porous enamel structure also, the EDX findings revealed a substantial decrease in the Ca & P content in addition to the F and Ca/P ratio. This could be related to the prolonged contact of the acidic NAC with the tooth surface so, the pH decreases, and more calcium is lost. These findings are consistent with Stephan, [44] who stated that after consuming sugar-containing foods and drinks, the pH of the dental plaque drops rapidly to a point which could induce demineralization of the dental enamel. Furthermore, these results agreed with Loesche, [45] who reported that if the pH of dental plaque drops to 5.0 -5.2, the salivary buffers are frustrated, and enamel starts to disintegrate as lactic acid permeates the tooth, expelling calcium and phosphorus ions from areas below the surface enamel.

Regarding carbon, nitrogen, and oxygen content, a noteworthy difference was found between all groups (P < 0.05) with a meaningful increase in group-S3. This significant increase may be the major reason for exacerbating the dissolution of hydroxyapatite crystals. The carbon ions can substitute for phosphate ions and, at high levels, can also replace the hydroxyl ions, causing the crystal less stable and increasing apatite dissolution [46]. This agreed with Sabel et al. [47] who observed markedly decreased levels of calcium and phosphorus along with higher levels of carbon and nitrogen in carious lesions in comparison to sound enamel. Moreover, this may be linked with the more porous primary enamel and its higher proclivity for dissolution. On the other hand, this result was inconsistent with Aidaros & Kamh, [48] who found a reduction of carbon ion percentage after the application of fluoride-based pits and fissures sealants containing nano-seashell & nano-pearl compared to the percentage increase in the control group.

Precautionary actions should be done in children consuming the effervescent formulations to avoid the possible negative impacts of dental erosion, including carious lesion formation & tooth sensitivity; this could be explained by the high sodium and/or potassium concentration and the bicarbonate presence in the effervescent preparations. Any effervescent must be permitted to entirely solubilize before its intake [49]. Also, in high-risk patients, it is advised not to leave the effervescent in the oral cavity for an extended period of time and parents should be advised to thoroughly clean the children’s mouths with water after consuming effervescent formulations [25].

The present study undoubtedly has a limitation of a small sample size whereas the research was carried out during the COVID-19 epidemic which limits the accessibility of a larger number of extracted teeth with the needed criteria. Moreover, this study was conducted in-vitro, which may not properly represent the clinical circumstances, and the in-vivo behavior of NAC could change. Future research should spotlight the titratable acidity, sugar content, and cariogenic ability of NAC. Another diagnostic tool might be suggested since SEM has inherent restrictions for the possible applications of in-vivo diagnosis of early demineralized lesions.

## Conclusion

Considering the findings and limitations of this study, NAC had a detrimental effect on initial enamel carious lesions in primary teeth also, the longer NAC was in contact with the teeth, the more enamel was lost, as well as the inorganic constituents.

This study can aid clinicians in their understanding of the caries process and how various medications affect enamel’s physical and chemical properties as well as its behavior during de- and remineralization.

## Data Availability

On reasonable request, the datasets utilized and/or analyzed during the present study are accessible from the corresponding author.
